# Immunorepertoire-based characterization of adaptive immunity in human malignant pleural effusion

**DOI:** 10.3389/fonc.2026.1825360

**Published:** 2026-04-28

**Authors:** Chuang-Xin Zhang, Xin-Ao Li, Yu-Peng Li, Kan Zhai

**Affiliations:** 1 Department of Respiratory and Critical Care Medicine, Beijing Institute of Respiratory Medicine and Beijing Chao-Yang Hospital, Capital Medical University, Beijing, China; 2 Clinical Center for Pleural Diseases, Capital Medical University, Beijing, China

**Keywords:** malignant pleural effusion, lung cancer, single-cell sequencing, T-cell receptor, B-cell receptor

## Abstract

**Background:**

Malignant pleural effusion (MPE) frequently occurs in patients with advanced cancer and is predominantly infiltrated by lymphocytes, particularly CD4^+^ T cells. However, the characteristics of adaptive immune cells, including T-cell receptor (TCR) and B-cell receptor (BCR) repertoires, in MPE remain largely unclear.

**Methods:**

In this study, we comprehensively characterized adaptive immune alterations in human lung adenocarcinoma-associated MPE and peripheral blood samples using single-cell RNA sequencing and single-cell V(D)J sequencing.

**Results:**

No significant difference in complementary determining region 3 length distribution was observed among adaptive immune cell subsets between MPE and blood samples. Within MPE microenvironment, regulatory T cells and regulatory B cells preferentially harbored specific V(D)J gene segments, while Th1/17 cells exhibited marked clonal expansion associated with metabolic adaptation, enhanced effector functions, and a propensity to recognize MPE-specific antigens. In addition, expanded cytotoxic CD8^+^ T cells displayed functional heterogeneity characterized by strong cytotoxic potential alongside an exhaustion signature. Notably, our findings underscore the functional importance of TCR complementary determining region 3 in determining MPE-specific antigen recognition. However, no significant clonotypic or functional changes were detected in the BCR repertoire.

**Conclusions:**

This study provides a TCR/BCR-based single-cell atlas of adaptive immune profiles in MPE, offering new insights into the immunopathogenesis of this malignancy-associated condition.

## 1 Introduction

Malignant pleural effusion (MPE) is a common and debilitating manifestation of either primary pleural malignancies or metastatic spread to the pleural cavity (1), occurring in up to 15% of all cancer patients (2). Although large-scale epidemiologic data remain limited, it is estimated that MPE accounts for more than 125,000 hospitalizations annually in the United States, with an inpatient mortality rate of 11.6%, indicating a high short-term mortality burden (3). MPE arises across diverse malignancies, with lung cancer–associated cases constituting the largest proportion (37.8%) (3). Clinically, patients with MPE frequently experience severe dyspnea, chest pain, and fatigue, accompanied by poor prognosis; notably, the median survival of lung cancer patients with MPE is approximately half that of those without effusion (4, 5). Current MPE management combines antitumor therapy with palliative interventions aimed at symptom relief; however, the immune composition of MPE and its complex interactions with tumor cells often impair therapeutic efficacy (6, 7).

The cellular composition of MPE is dominated by lymphocytes, particularly CD4^+^ T cells (8, 9). The remarkable complexity and adaptability of the adaptive immune system are primarily governed by the vast diversity of T-cell receptors (TCRs) and B-cell receptors (BCRs) (10). These immunoglobulin molecules, expressed on the surfaces of T and B cells, respectively, enable antigen recognition and subsequent immune activation. During T- and B-cell development, TCRs and BCRs are generated via somatic recombination through the V(D)J mechanism, whereby one variable (V), one diversity (D), and one joining (J) gene segment are selected and recombined, thereby contributing to the immense diversity of antigen receptor repertoires (11). The variable region of T-cell receptor β-chain (TRB) or immunoglobulin heavy-chain (IGH) is the most essential site for antigen binding. Within these regions, three complementary determining regions (CDRs), including CDR1, CDR2, and CDR3, determine the specificity of antigen binding. Among them, CDR3, which spans the V(D)J junction, is the most variable region and directly mediates antigen contact, thus determining receptor specificity (12). To facilitate the prediction of antigen-specific TCRs and predict specific antigen, the improved Grouping of Lymphocyte Interactions by Paratope Hotspots version 2 (GLIPH2) algorithm was developed, enabling highly accurate inference of TCR–antigen specificity relationships (13). Accordingly, TCR and BCR repertoire characteristics are closely associated with a range of immune-related, infectious, and malignant diseases.

Given the key role of TRB and IGH, compared with TRA and IGL, in epitope binding, we focused our analysis on TRB and IGH repertoires in MPE from lung adenocarcinoma patients. In our previous study, we characterized diverse immune phenotypes in MPE using single-cell RNA sequencing combined with paired single-cell TCR-seq and BCR-seq (14). Building upon these data, we analyzed clonal diversity within T- and B-cell repertoires, assessed clonal expansion, and examined differential gene expression across adaptive immune cell subsets. Moreover, we predicated MPE-specific antigens recognized by T cells. Together, these analyses highlight the utility of TCR/BCR profiling for elucidating T and B cell–mediated immune responses in the pathogenesis of MPE.

## 2 Materials and methods

### 2.1 Study participants, raw sequencing data acquisition

Matched MPE and peripheral blood samples were collected from five patients with lung adenocarcinoma. The demographic and sample characteristics of these participants were detailed in our earlier publication (14). Raw data for single-cell mRNA sequencing, TCR sequencing, and BCR sequencing were used and could be obtained from the Genome Sequence Archive for Human (HRA000153) and the Gene Expression Omnibus (GSE185058). Cell filtering, quality control, and clustering procedures were performed as described in our previous study (14). Ethical review and approval were waived for this study because the original data analyzed were retrieved from publicly accessible databases.

### 2.2 TCR and BCR repertoire analysis

Immune repertoire (IR) analyses were conducted using Cell Ranger for sequence alignment, quality filtering, barcode assignment, and Unique Molecular Identifier quantification. Cells containing a unique productive TCRβ or BCR heavy-chain sequence were retained for further analyses under the following criteria: (i) all T or B cells possessing only one productive TRB or IGH chain were included; (ii) for T cells with multiple productive β chains, only the dominant one was retained; and (iii) similarly, for B cells expressing more than one productive heavy chain, only the predominant chain was analyzed. Clonal enrichment was defined as the detection of identical β or heavy-chain sequences in at least two cells within the same cell population.

The CDR3 distribution profile was assessed using mean values and statistical descriptors typical of Gaussian-like distributions, including kurtosis and skewness (15). Kurtosis reflects the concentration of values near the mean relative to the distribution tails, representing the degree of peakedness, while skewness measures asymmetry around the mean (16). Diversity and clonotypic expansion were quantified using the Diversity 50 (D50) index, which represents the fraction of unique CDR3 sequences accounting for 50% of all observed sequences (17).

The somatic hypermutation (SHM) rate was determined by quantifying nucleotide substitutions between the inferred germline genes and experimentally identified BCR sequences. Mutation events were subsequently calculated with the package Shazam (v1.3.1) in R (v4.2.0) to estimate the SHM level for each individual BCR sequence.

### 2.3 Differentially expressed gene and pathway analyses

Differentially expressed gene (DEG) analysis between two cell populations was performed using the R package limma (v3.62.2), with statistical significance determined after Benjamini–Hochberg multiple testing correction at *P*_adj_ < 0.05. Functional enrichment analysis was conducted using the R package clusterProfiler (v4.14.6). Gene lists were derived from biological process terms in the Gene Ontology (GO) database. Kyoto Encyclopedia of Genes and Genomes (KEGG) pathway enrichment analysis was performed on DEGs. Metabolic-related pathways were selected according to KEGG pathway classification. To ensure statistical reliability, pathways with *P*_adj_ < 0.05 were defined as significantly enriched.

### 2.4 Diffusion map analysis

Trajectory analysis based on diffusion map dimensionality reduction was performed using the R package Destiny (v3.14.0). Cells preprocessed with the R package Seurat (v4.3.0) were used as input, and the first 30 principal components were selected as the feature matrix. Euclidean distance was used to compute cell–cell similarities. Diffusion maps were generated by calculating the eigenvalues and eigenvectors of the diffusion operator, where eigenvalues represent the variance explained by each diffusion component and eigenvectors define the coordinates of cells in diffusion space. The first three diffusion components (DC1–DC3) were retained for downstream analysis.

### 2.5 TCR sequence clustering by GLIPH2

GLIPH2 is an algorithm designed to cluster large numbers of TCRs into groups that share common antigen specificities (13). Using GLIPH2 algorithm, clusters were identified by global similarity and local similarity. Significant clusters were considered based on the following parameters: Fisher score < 0.05 and shared subjects ≥ 2. The GLIPH2 algorithm, reference CDR3β sequences, and a tutorial are publicly available (http://50.255.35.37:8080).

### 2.6 Statistical analysis

Statistical significance was assessed using the Mann-Whitney U test or t test, as appropriate. All statistical analyses were conducted using GraphPad Prism (version 2.0), and two-tailed *P*-values < 0.05 were considered statistically significant.

## 3 Results

### 3.1 Defining T- and B-cell subsets with paired T- and B-cell receptor repertoires

We performed scRNA-seq on mononuclear cells isolated from MPE and matched peripheral blood samples obtained from five lung cancer patients. After sequencing quality control steps, we obtained 44,545 T cells and 4,361 B cells. Because the TCR-seq data for T cells were previously reported (18), the corresponding BCR-seq data for B cells from each patient are presented in **Supplementary Table 1**, with an total of 39,308,047 read pairs. Integrating TCR-seq and BCR-seq data, we identified 37,009 T cells and 3,180 B cells with uniquely paired productive TCR β chains or productive BCR heavy chains, respectively (14). Among these, we detected 22,717 CD4^+^ T cells (8,590 naïve CD4^+^ T cells; 10,863 Th1/17 cells; 2,208 regulatory T cells (Tregs); and 1,056 Tfh cells), 14,284 CD8^+^ T cells (1,029 naïve CD8^+^ T cells; 9,923 cytotoxic CD8^+^ T cells; and 3,332 exhausted CD8^+^ T cells), 8 proliferating T cells, 1,816 naïve B cells, 1,243 regulatory B cells (Bregs), and 121 plasma cells across MPE and blood samples (**Supplementary Table 2**).

### 3.2 CDR3 length distributions

**Figure 1:**
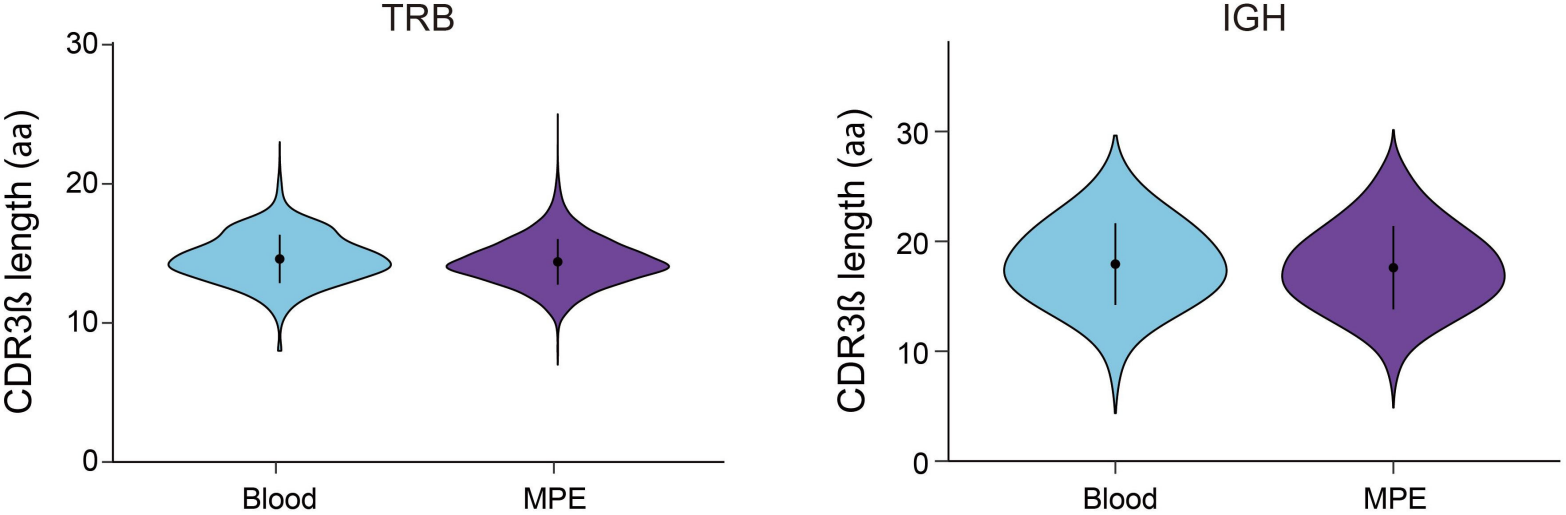
CDR3β length distribution in T and B cells from MPE and blood. All *P* > 0.05, determined by Mann-Whitney U test.

CDR3 length and composition play critical roles in adaptive immune recognition and antigen-specific T-cell responses, as longer CDR3 regions allow for greater sequence diversity. We next examined the CDR3 length distribution of unique TRB and IGH transcripts in MPE and blood samples. The CDR3 length of TRB and IGH, and length profile distributions in total T cells (ranging from 7 to 25 nucleotides vs. 8 to 23 nucleotides) and in total B cells (ranging from 7 to 28 nucleotides vs. 7 to 27 nucleotides) were similar between MPE and blood (**Figure 1**). However, for other T- and B-cell subsets, both TRB and IGH CDR3 length, and length variability (as indicated by the kurtosis index) were similar, and distributions remained symmetric according to skewness measurements between MPE and blood (**Supplementary Figures 1A-C**).

### 3.3 Usage patterns of variable and joining gene segments in TCR and BCR repertoires

To examine differences of IR composition between MPE and blood, we compared TRB and IGH V and J gene usage in T and B cells, respectively; and identified several segments with significantly different usage frequencies. Usage patterns of TRBV, TRBJ, and TRBV–TRBJ combinations were analyzed across all patients and are illustrated in **Supplementary Figures 2A, B**. Comparative analysis of TRB V and J gene segment usage among different T-cell subsets revealed significant differences between MPE and blood. For TRBV genes (**Table 1**), several segments, including TRBV5-1, TRBV10-3, TRBV12-3, TRBV12-4, TRBV20-1, and TRBV24-1, were significantly enriched in Treg cells in MPE samples relative to blood samples, whereas TRBV11–3 frequency was reduced in naïve CD4^+^ T cells. Among CD8^+^ T-cell subsets, only TRBV12–3 was increased in cytotoxic CD8^+^ T cells in MPE compared with blood. Analysis of TRBJ segments revealed increased TRBJ1–5 and TRBJ2–3 usage in Tregs from MPE, while TRBJ2–7 usage was decreased in cytotoxic CD8^+^ T cells. IGH chain (IGHV and IGHJ) gene usage was examined across all patients (**Supplementary Figures 2C, D**). The results revealed that Bregs exhibited relatively high IGHV1-2, IGHV4-61, and IGHV6–1 usage in MPE as compared with paired blood samples (**Table 1**). In addition, IGHJ3 and IGHJ6 usage was decreased in naïve B cells from MPE samples, whereas IGHJ3, IGHJ5, and IGHJ6 usage was increased in Bregs. Conversely, IGHJ4 and IGHJ5 usage was reduced in plasma cells from MPE samples relative to those in blood. These findings indicate the presence of a distinct IR within the MPE microenvironment, in which Tregs and Bregs preferentially harbor specific V/J gene segments.

**Table 1:** The differences in the usage of V and J genes between MPE and paired blood cells*.

Cell type	V gene (Increased)	V gene (Decreased)	J gene (Increased)	J gene (Decreased)
T cell				
Naïve CD4^+^ T	NA	TRBV11-3	NA	NA
Treg	TRBV5-1, TRBV10-3, TRBV12-3, TRBV12-4, TRBV20-1, TRBV24-1	NA	TRBJ1-5, TRBJ2-3	NA
Cytotoxic CD8^+^ T	TRBV12-3	NA	NA	TRBJ2-7
B cell				
Naïve B cell	NA	NA	NA	IGHJ3, IGHJ6
Bregs	IGHV1-2, IGHV4-61, IGHV6-1	NA	IGHJ3, IGHJ5, IGHJ6	
Plasma	NA	NA	NA	IGHJ4, IGHJ5

*P < 0.05 in each comparison, determined by paired t test; MPE, malignant pleural effusion; NA, not available.

### 3.4 Clonal expansion in MPE patients

**Figure 2:**
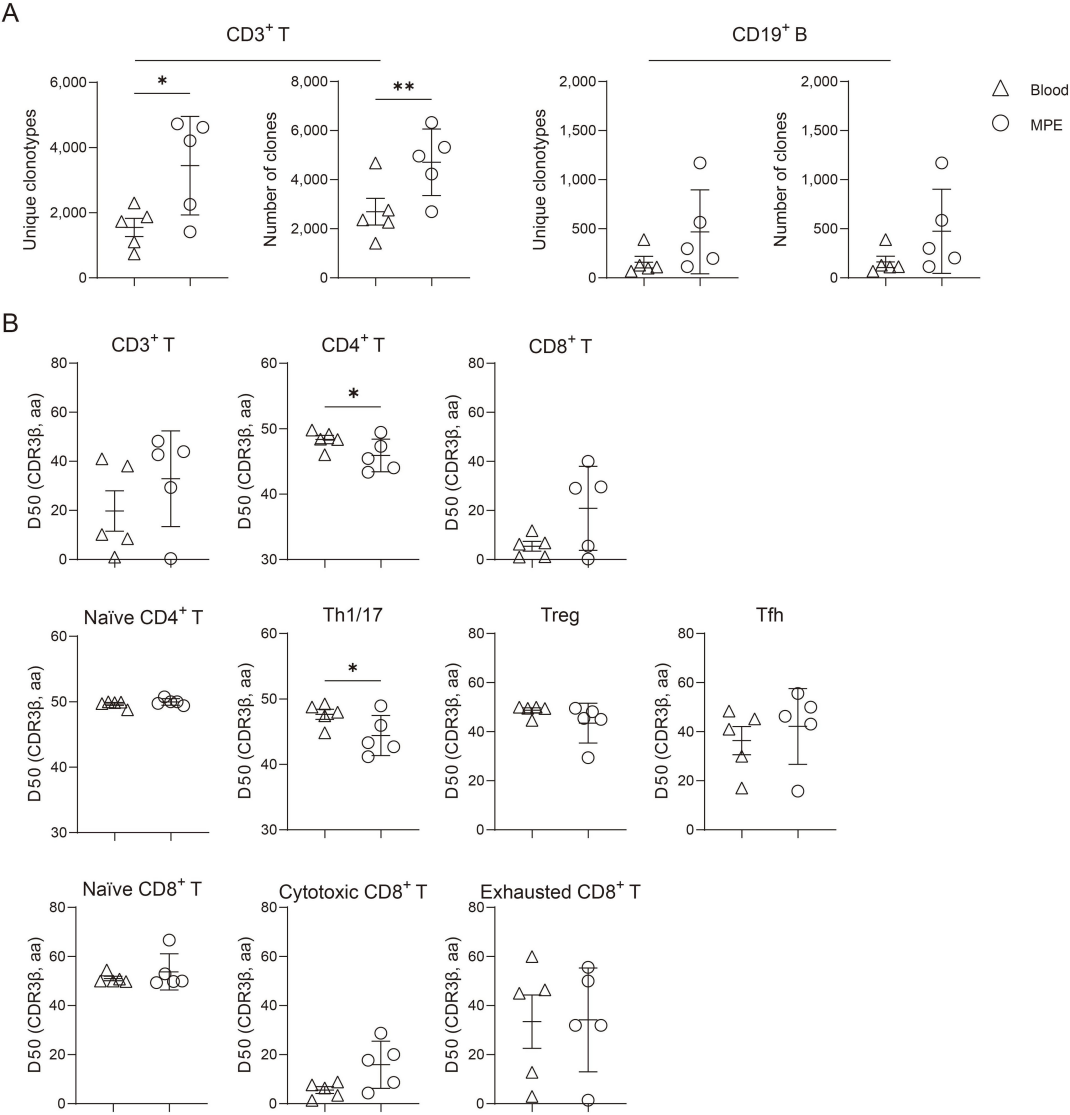
Characteristics of TCR and BCR repertoires in MPE and blood. **(A)** Comparison of the numbers of unique clonotypes and total clone counts between MPE and blood. **(B)** Diversity 50 (D50) index of TCR repertoires across T cell subsets from MPE and blood. The D50 index is defined as the proportion of unique CDR3 sequences required to account for 50% of the total repertoire. A smaller D50 value indicates that fewer clonotypes contribute to 50% of the total number clones, reflecting a higher degree of clonal expansion. Data are presented as means ± SEM. *P < 0.05, **P < 0.01, determined by paired t test.

We quantified the number and diversity of clones in both MPE and blood samples and observed significant differences, with higher clonal counts in MPE (**Figure 2A**). As clonal expansion patterns may vary across microenvironments, we further characterized T- and B-cell subsets clonal expansion using the D50 index based on CDR3 sequences. Lower D50 values correspond to greater clonal expansion. The D50 index of total CD4^+^ T cells was significantly lower in MPE than in blood, suggesting enhanced clonal expansion (**Figure 2B**). While most T-cell subsets displayed comparable D50 indices between MPE and blood, the D50 of Th1/17 cells was significantly reduced in MPE (*P* = 0.041), indicating preferential expansion within the pleural microenvironment.

**Figure 3:**
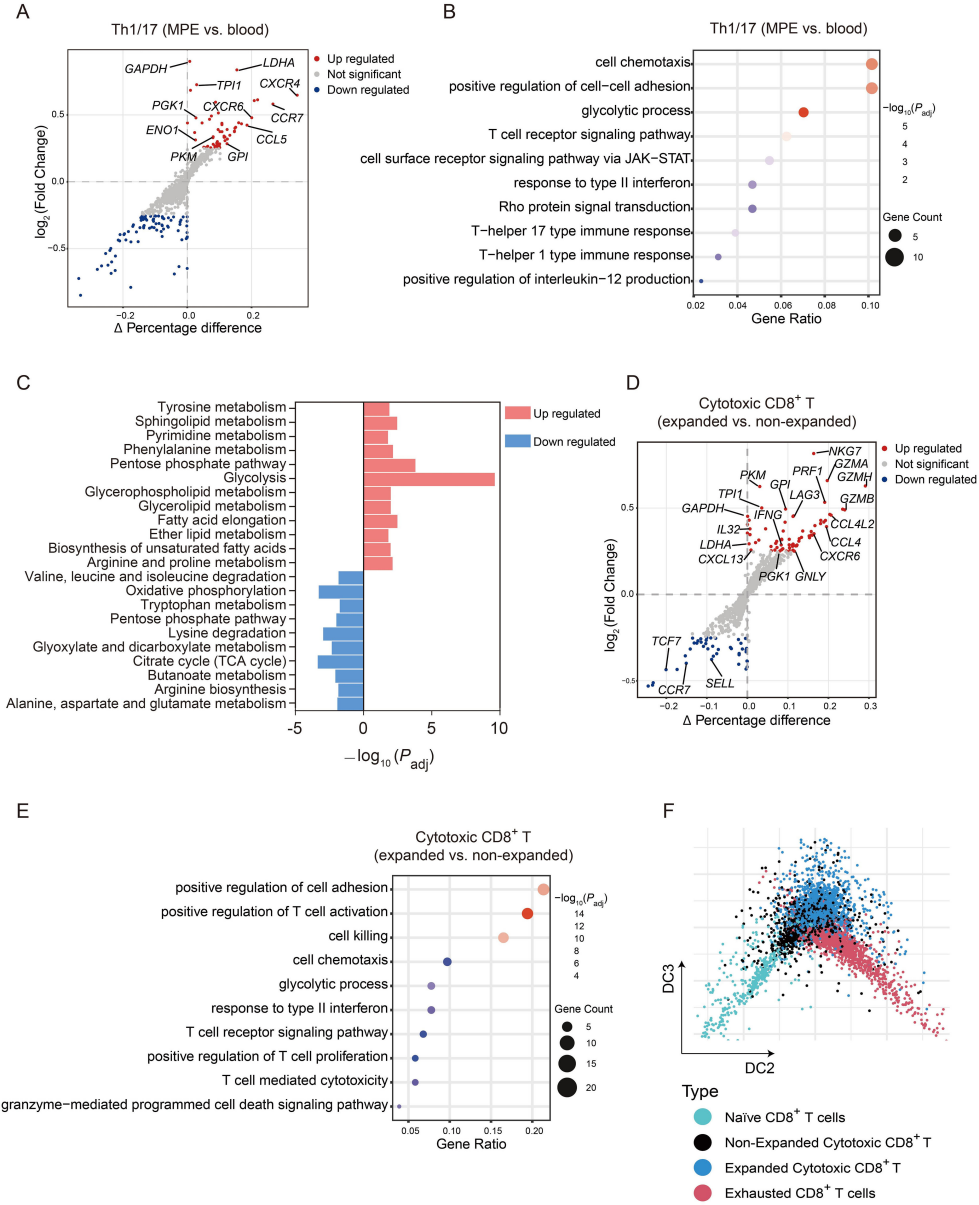
DEGs, pathways, and diffusion map trajectory analysis of Th1/17 and cytotoxic CD8^+^ T cell subsets. **(A)** Modified volcano plot showing DEGs between MPE-derived and blood–derived Th1/17 cells **(B)** Bubble chart illustrating the top ten GO terms enriched among DEGs identified in the Th1/17 cell comparison. **(C)** Metabolic-related KEGG enrichment analysis between MPE-derived and blood–derived Th1/17 cells. **(D)** Modified volcano plot depicting DEGs between expanded and non-expanded cytotoxic CD8^+^ T cells. **(E)** Bubble chart showing the top ten GO terms enriched among DEGs identified in the cytotoxic CD8^+^ T cell comparison. **(F)** Diffusion map trajectory analysis illustrating the developmental continuum of CD8^+^ T cell subsets along the DC2 axis.

We next compared the transcriptional profiles of MPE-derived and blood-derived Th1/17 cells. Differential expression analysis (log2|FC| > 0.25, *P*_adj_ < 0.05) identified 62 upregulated and 80 downregulated genes in MPE-derived Th1/17 cells (**Figure 3A**). GO enrichment analysis revealed that upregulated genes were involved in biological processes related to T-cell function and activation (e.g., TCR signaling and Th1/Th17 responses), signal transduction, and IL-12 production, indicating enhanced effector activity in the pleural microenvironment (**Figure 3B**). The top upregulated genes in MPE-derived Th1/17 cells were associated with glycolysis (*GAPDH*, *LDHA*, *TPI1*, *PGK1*, *ENO1*, *GPI*, and *PKM*), suggesting metabolic reprogramming toward aerobic glycolysis to sustain activation under hypoxic conditions. KEGG enrichment analysis revealed a significant enrichment of glycolysis-related pathways, together with a reciprocal reduction in the enrichment of oxidative phosphorylation signatures in MPE-derived Th1/17 cells compared with blood-derived Th1/17 cells (**Figure 3C**). Collectively, these findings support the conclusion that MPE-derived Th1/17 cells undergo metabolic reprogramming toward aerobic glycolysis, which may contribute to maintain their activation state in hypoxic MPE microenvironments. Additionally, the expression levels of genes involved in chemotaxis and adhesion (*CCL5*, *CXCR4*, *CCR7*, and *CXCR6*) were elevated, implying enhanced migratory and tissue-resident capacities. Conversely, blood-derived Th1/17 cells showed higher expression of *TAGAP* and *DOCK10*, suggesting augmented Rho signaling activity, consistent with differentially active signaling pathways (**Figure 3A**).

Within the same MPE microenvironment, different immune cell subsets exhibited heterogeneous clonal expansion dynamics, and cells with significant expansion displayed distinct transcriptional characteristics. We observed that cytotoxic CD8^+^ T cells were markedly expanded in both MPE and blood, with no significant differences between the two compartments (**Figure 2B**). To further investigate functional differences, we compared expanded cytotoxic CD8^+^ T cells (clone number ≥ 2) with non-expanded cytotoxic CD8^+^ T cells (clone number = 1). Differential expression analysis (log2|FC| > 0.25, *P*_adj_ < 0.05) identified 67 upregulated and 44 downregulated genes in expanded cells (**Figure 3D**). Expanded cytotoxic CD8^+^ T cells showed increased expression of genes associated with cytotoxic activity, including *NKG7*, *GZMA*, *GZMH*, *GZMB*, *PRF1*, *IL32*, *GNLY*, and *IFNG*; chemokines and receptors such as *CCL4L2*, *CCL4*, *CXCL13*, and *CXCR6*; glycolysis-related genes such as *GAPDH*, *PKM*, *TPI1*, *LDHA*, *GPI*, and *PGK1*; and the exhaustion marker *LAG3*. Correspondingly, pathway enrichment analysis revealed upregulation of biological processes related to cell killing, T-cell-mediated cytotoxicity, chemotaxis and cell adhesion, glycolytic metabolism, and response to type II interferon (**Figures 3D, E**). In contrast, non-expanded cytotoxic CD8^+^ T cells expressed higher levels of *CCR7*, *TCF7*, and *SELL*, indicative of a more stem−like and naïve phenotype state (19), whereas expanded cytotoxic CD8^+^ T cells exhibited lower expression of these genes with a reduced stem-like and circulating naïve phenotype. Diffusion map trajectory analysis further illustrated the developmental continuum of CD8^+^ T subsets along the DC2 axis: expanded cytotoxic CD8^+^ T cells were positioned closer to exhausted CD8^+^ T cells, supporting their transitional state toward exhaustion (**Figure 3F**); while, exhausted CD8^+^ T cells represented the terminal differentiation state within the MPE microenvironment. Collectively, these results suggest that expanded cytotoxic CD8^+^ T cells undergo metabolic and transcriptional adaptation to enhance their effector function and energy production, while also acquiring features of exhaustion in the MPE microenvironment.

For the analysis of B cells, the expansion of various B-cell subsets did not exhibit any significant differences between MPE and blood samples, nor were there no significant differences among the B-cell subsets within MPE (**Supplementary Figure 3A**). To further investigate the characteristics of the BCR repertoire in MPE, we analyzed the levels of SHM in B-cell subsets. Consistently, no significant differences in SHM levels were observed between MPE-derived B cells and blood-derived B cells (**Supplementary Figure 3B**).

### 3.5 Identification of clusters of similar TCRβ sequences in MPE patients

**Figure 4:**
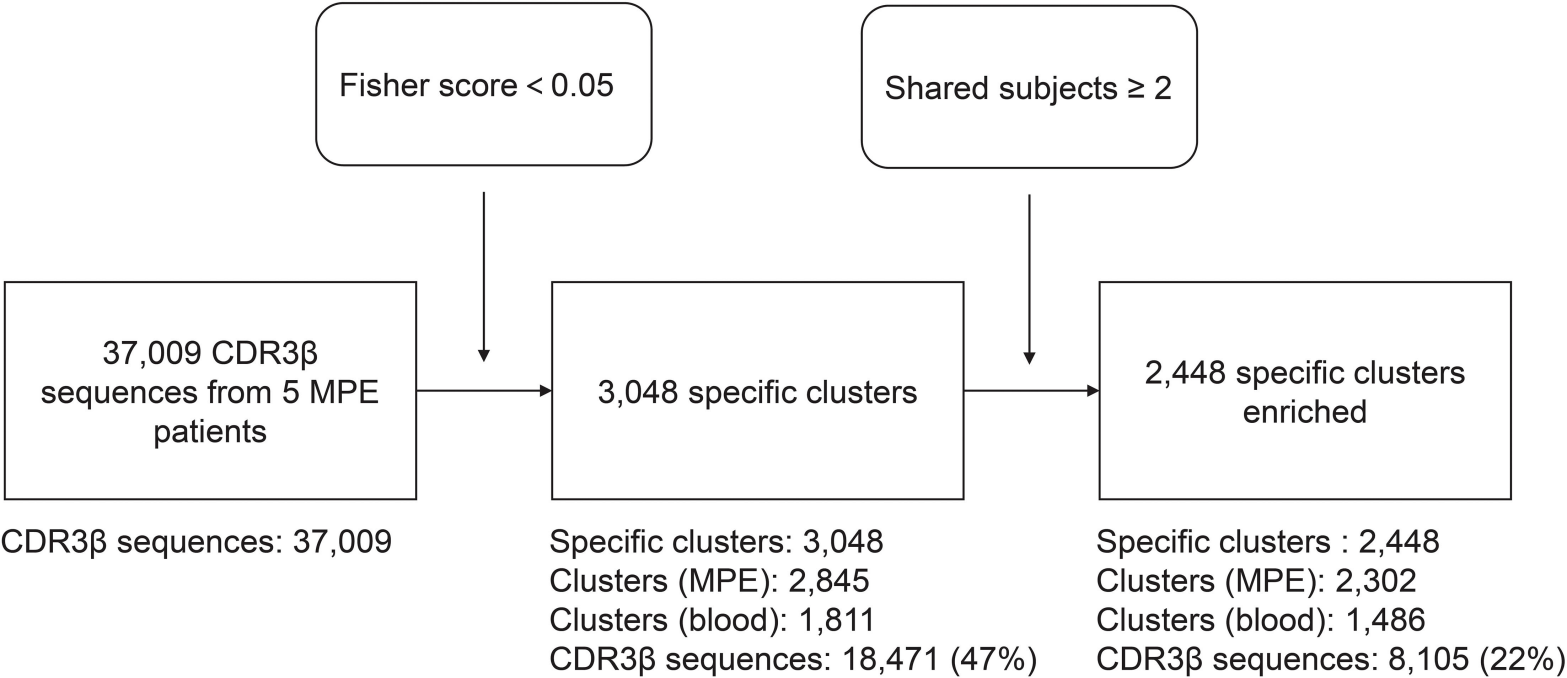
Workflow of GLIPH2 analysis for identifying TCR CDR3 similarity.

To identify specific TCRβ sequences potentially involved in MPE-associated immune responses, we applied the GLIPH2 algorithm to predict shared TCR sequence motifs and cluster TCRs recognizing the same or similar epitopes. The workflow of the GLIPH2 analysis is summarized in **Figure 4**. GLIPH2 analysis grouped a total of 37,009 cells containing 24,134 TCR clonotypes from MPE and blood into 2,448 TCR specificity groups, based on comparison with external TCR datasets (Fisher score < 0.05; shared subjects ≥ 2). Among these, 23,532 cells comprising 17,173 clonotypes were grouped into 2,303 clusters in MPE samples, while 13,477 cells with 7,725 clonotypes formed 1,486 clusters in blood. The frequency of grouped cells was significantly higher in MPE than in blood (P ≤ 0.001), indicating enrichment of T-cell groups likely recognizing MPE-specific antigens in MPE patients.

We next focused on the GLIPH2 clustering results for Th1/17 cells, aiming to determine whether their clonal expansion in MPE was associated with cell with MPE-specific TCRs. A total of 172 highly expanded Th1/17 clonotypes (clone number ≥ 3) from 684 cells were identified within the MPE-derived Th1/17 cells. After matching these 172 clonotypes with the GLIPH2 analysis results (Fisher score < 0.05; shared subjects ≥ 2), 33 clonotypes were retained, corresponding to 46 TCR clusters. Similar TCRβ sequences may represent T cells recognizing identical or related antigens. Among these 46 TCR groups, the clusters “S%TGGNTE,” “SLG%E,” “SLTGG%TE,” and “S%GET” were detected in all five patients (**Table 2**), suggesting that these individuals shared comparable TCRβ sequences, which may indicate recognition of similar antigen. These shared motifs may further represent TCRs involved in MPE-specific immune responses and contribute to the immunopathogenesis of MPE.

**Table 2:** S%TGGNTE, SLG%E, SLTGG%TE, and S%GET pattern clusters of similar CDR3 by grouping of lymphocyte interactions by paratope hotspots version 2*.

Pattern	Fisher _score	Number _subject	Number _unique_CDR3	Type	TRB	V	J
S%TGGNTE	0.0048	5	4	global-S%TGGNTE	CASSLTGGNTEAFF	TRBV7-9	TRBJ1-1
S%TGGNTE	0.0048	5	4	global-S%TGGNTE	CASSLTGGNTEAFF	TRBV7-9	TRBJ1-1
S%TGGNTE	0.0048	5	4	global-S%TGGNTE	CASSLTGGNTEAFF	TRBV14	TRBJ1-1
SLG%E	0.0036	5	11	global-SLG%E	CASSLGSEAFF	TRBV12-3	TRBJ1-1
SLG%E	0.0036	5	11	global-SLG%E	CASSLGSEAFF	TRBV12-3	TRBJ1-1
SLTGG%TE	0.0021	5	4	global-SLTGG%TE	CASSLTGGNTEAFF	TRBV7-9	TRBJ1-1
SLTGG%TE	0.0021	5	4	global-SLTGG%TE	CASSLTGGNTEAFF	TRBV7-9	TRBJ1-1
SLTGG%TE	0.0021	5	4	global-SLTGG%TE	CASSLTGGNTEAFF	TRBV14	TRBJ1-1
S%GET	0.0046	5	8	global-S%GET	CASSLGETQYF	TRBV5-6	TRBJ2-5
S%GET	0.0046	5	8	global-S%GET	CASSLGETQYF	TRBV5-1	TRBJ2-5
S%GET	0.0046	5	8	global-S%GET	CASSLGETQYF	TRBV13	TRBJ2-5
S%GET	0.0046	5	8	global-S%GET	CASSLGETQYF	TRBV7-9	TRBJ2-5
S%GET	0.0046	5	8	global-S%GET	CASSLGETQYF	TRBV7-2	TRBJ2-5
S%GET	0.0046	5	8	global-S%GET	CASSLGETQYF	TRBV7-2	TRBJ2-5
S%GET	0.0046	5	8	global-S%GET	CASSLGETQYF	TRBV7-6	TRBJ2-5

*%, amino acid positions in the TCRs within the clusters that are available for substitution; global, global similarity.

## 4 Discussion

MPE is a lymphocyte-predominant effusion in which T and B lymphocytes play central roles in disease progression and immune evasion. The IR reflects the entire spectrum of TCRs and BCRs, providing a snapshot of antigen-driven and bystander lymphocyte activity at specific disease stages (20). However, an integrated characterization of T and B cells that links matched TCR and BCR repertoires with transcriptional profiles is still lacking. Such integration is critical for deciphering the molecular principles governing antigen recognition by TCRs and BCRs. Although the heterogeneity of the TCR repertoire has been previously investigated in MPE from lung cancer patients (21), direct comparisons between MPE and paired blood samples remain absent, and BCR characteristics have not been reported. In this study, we employed single-cell technologies, including scRNA-seq, scTCR-seq, and scBCR-seq, to comprehensively delineate the compositional and functional adaptations of T- and B-cell subsets in human MPE.

Although CDR3 length is associated with immune functionality in various diseases, we observed no significant differences in CDR3 length distribution between MPE and matched blood samples. Identifying TCR sequences linked to immune responses is critical to understanding MPE pathogenesis. Here, we analyzed V/J segment usage across adaptive immune cell subsets, as V(D)J recombination generates repertoire diversity and clonality, with specific usage patterns reflecting clonal constraints. We found skewed usage of individual V and J genes in certain cell subsets within MPE compared with blood, suggesting that unique V–J pairing patterns may represent MPE-associated adaptive immune responses. Notably, Tregs and Bregs from MPE samples exhibited higher frequencies of TRBV (TRBV5-1, TRBV10-3, TRBV12-3, TRBV12-4, TRBV20-1, and TRBV24-1), TRBJ (TRBJ1–5 and TRBJ2-3), IGHV (IGHV1-2, IGHV4-61, and IGHV6-1), and IGHJ (IGHJ3 and IGHJ6) usage than other adaptive immune subsets. These findings raise the possibility that regulatory lymphocytes in MPE recognize distinct antigenic determinants relevant to MPE pathogenesis, at least from a CDR3 usage perspective. Moreover, these V and J segments have been previously implicated in tumor- and infection-associated immune responses, supporting the validity of our observations; for example, TRBV5-1 (22–24), TRBV12-4 (25, 26); TRBV20-1 (27–31), and IGHV1-2 (32).

Previous studies have shown that Th1, Th17, Treg, Th9, and Th22 cells are increased in human MPE compared with paired blood samples (33). Although Th9 and Th22 subsets were not identified in our dataset, we observed reduced CDR3 diversity within Th1/17 cells in MPE, indicative of clonal expansion likely driven by antigen-specific selection pressures. This observation provides a plausible explanation for the increased abundance of Th1/17 cells in MPE. In lung cancer-associated MPE, Th17 cell numbers positively correlate with Th1 cell frequencies (34), and our results reveal that MPE-derived Th1/17 cells exhibit distinct transcriptional and functional profiles compared with their blood-derived counterparts. IR analysis underscores the profound influence of the MPE microenvironment in shaping Th1/17 cell identity and function. The observed transcriptional and metabolic reprogramming suggests that local microenvironmental cues enhance effector capacity, migratory potential, and responsiveness to inflammatory signals. This adaptation likely reflects selective pressures within MPE that favor energetically demanding, functionally specialized T-cell subsets. In contrast, blood-derived Th1/17 cells displayed features of a less activated, metabolically quiescent phenotype. Collectively, these findings suggest that the MPE microenvironment enhances Th1/17 cell metabolic fitness and effector functionality, offering potential therapeutic insights for optimizing T-cell responses in MPE-associated malignancies.

Relative to other subsets, cytotoxic CD8^+^ T cells exhibited significant clonal expansion in both MPE and blood, with comparable expansion levels across compartments. Our IR-based analysis indicates that clonal expansion of cytotoxic CD8^+^ T cells in MPE is tightly coupled with functional and metabolic reprogramming. Expanded clones expressed high levels of cytotoxic molecules and glycolytic enzymes, suggesting that antigen-driven proliferation is accompanied by enhanced effector function and metabolic adaptation. The concurrent upregulation of exhaustion markers further implies that persistent antigenic stimulation within MPE drives a dual trajectory of heightened cytotoxicity and progressive functional exhaustion. Conversely, non-expanded CD8^+^ T cells retained expression of naïve-associated genes, highlighting clonal selection as a key determinant of T-cell fate in the pleural environment. These results suggest that clonal expansion is a major mechanism by which the MPE microenvironment shapes the functional heterogeneity of cytotoxic CD8^+^ T cells.

Predicting T-cell reactivity to specific antigens remains a major challenge in TCR repertoire research. Because direct TCR–antigen binding prediction is highly complex, current computational strategies focus on identifying groups of TCRs that share conserved CDR3 motifs (35, 36). Using GLIPH2, we identified multiple TCR specificity groups enriched in MPE, reflecting the complexity of local antigen-driven immune responses. Of particular note, several specificity clusters were significantly enriched among MPE-derived Th1/17 cells, including TCR clusters found in all five patients. Clusters such as “S%TGGNTE,” “SLG%E,” “SLTGG%TE,” and “S%GET” suggest the recognition of similar antigens in MPE. These shared motifs may reflect TCRs involved in MPE-specific immune responses. However, further studies are needed to identify the antigens recognized by these functionally similar clusters and to elucidate the underlying mechanisms.

In the present study, we analyzed clonal expansion and SHM in MPE-derived and blood-derived B cells, and both analyses revealed no statistically significant differences. Data on class switch recombination in these patients were reported in our previous study, which also showed no significant differences between the groups (14). Based on these findings, we infer that B cells in the MPE microenvironment are more likely to function primarily through helper or regulatory mechanisms that modulate other immune cells. A previous study demonstrated that Bregs within MPE can promote the differentiation of Tregs (37), further supporting the notion that B cells in MPE play critical immunoregulatory roles independent of antigen-dependent processes such as clonal expansion, SHM, or class switch recombination.

Our study has several limitations. The sample size was relatively small, comprising only five MPE patients with lung adenocarcinoma, which may limit statistical power. Larger longitudinal cohorts are needed to delineate disease-specific clusters and dynamic IR changes associated with MPE progression. Additionally, potential confounding factors influencing IR characteristics warrant further investigation. Despite these limitations, our study provides a comprehensive overview of TCR and BCR repertoires across adaptive immune cell subsets in MPE. We identified preferential V/J segment and CDR3 usage patterns predominantly in regulatory subsets, while Th1/17 cells exhibited extensive clonal expansion, potential MPE-specific antigen binding, and enhanced effector activity compared with blood-derived counterparts. Furthermore, the MPE microenvironment profoundly shaped the functional heterogeneity and metabolic programming of cytotoxic CD8^+^ T cells. Collectively, our findings reveal novel features of IR-based adaptive immunity in MPE pathogenesis and may inform the future development of immune-targeted therapeutic strategies.

## 5 Conclusions

In summary, this study delineates the single-cell immune repertoire landscape of MPE in lung adenocarcinoma, revealing distinct adaptive immune remodeling within the pleural microenvironment. We identified skewed V/J gene usage in Tregs, pronounced Th1/17 clonal expansion with metabolic reprogramming, and cytotoxic CD8^+^ T cells exhibiting both enhanced effector activity and exhaustion signatures. The enrichment of shared TCR clusters predicted by GLIPH2 implies the presence of common MPE-related antigens driving local immune selection.

Overall, MPE constitutes a unique immune microenvironment shaped by persistent antigenic stimulation and functional adaptation of T cells and B cells. Integrating repertoires with transcriptional signatures advances understanding of MPE-specific antigen responses and provides a foundation for future studies aimed at antigen characterization and the development of targeted immunotherapies for pleural malignancy.

## Data Availability

The datasets presented in this study can be found in online repositories. The names of the repository/repositories and accession number(s) can be found in the article/**Supplementary Material**.
