# Correction: Immunorepertoire-based characterization of adaptive immunity in human malignant pleural effusion

**DOI:** 10.3389/fonc.2026.1879413

**Published:** 2026-05-19

**Authors:** Frontiers Production Office

**Keywords:** malignant pleural effusion, lung cancer, single-cell sequencing, T-cell receptor, B-cell receptor

The **Acknowledgments** was incorrect in the article. The statement has been corrected to read:

“We thank all the participants and their families.”

The original version of this article has been updated.

