# Supplementary material for: Immunorepertoire-based characterization of adaptive immunity in human malignant pleural effusion
Source: Front Oncol. 2026 Apr 28;16:1825360. doi: 10.3389/fonc.2026.1825360 (PMC13160786; doi:10.3389/fonc.2026.1825360)
Supplement: Supplementary file 1 [file DataSheet1.pdf]

Supplementary Material

Immunorepertoire-based characterization of adaptive immunity in human malignant pleural effusion

Chuang-Xin Zhang[1,2,†], Xin-Ao Li[1,2,†], Yu-Peng Li[1,2], Kan Zhai[1,2,*]

[1] Department of Respiratory and Critical Care Medicine, Beijing Institute of Respiratory Medicine and Beijing Chao-Yang Hospital, Capital Medical University, Beijing, 100020, China

[2] Clinical Center for Pleural Diseases, Capital Medical University, Beijing, 100020, China

[†]Chuang-Xin Zhang and Xin-Ao Li contributed equally to this work

*** Correspondence:**
Kan Zhai
zhaikan@ccmu.edu.cn

Supplementary files include three figures and two tables.

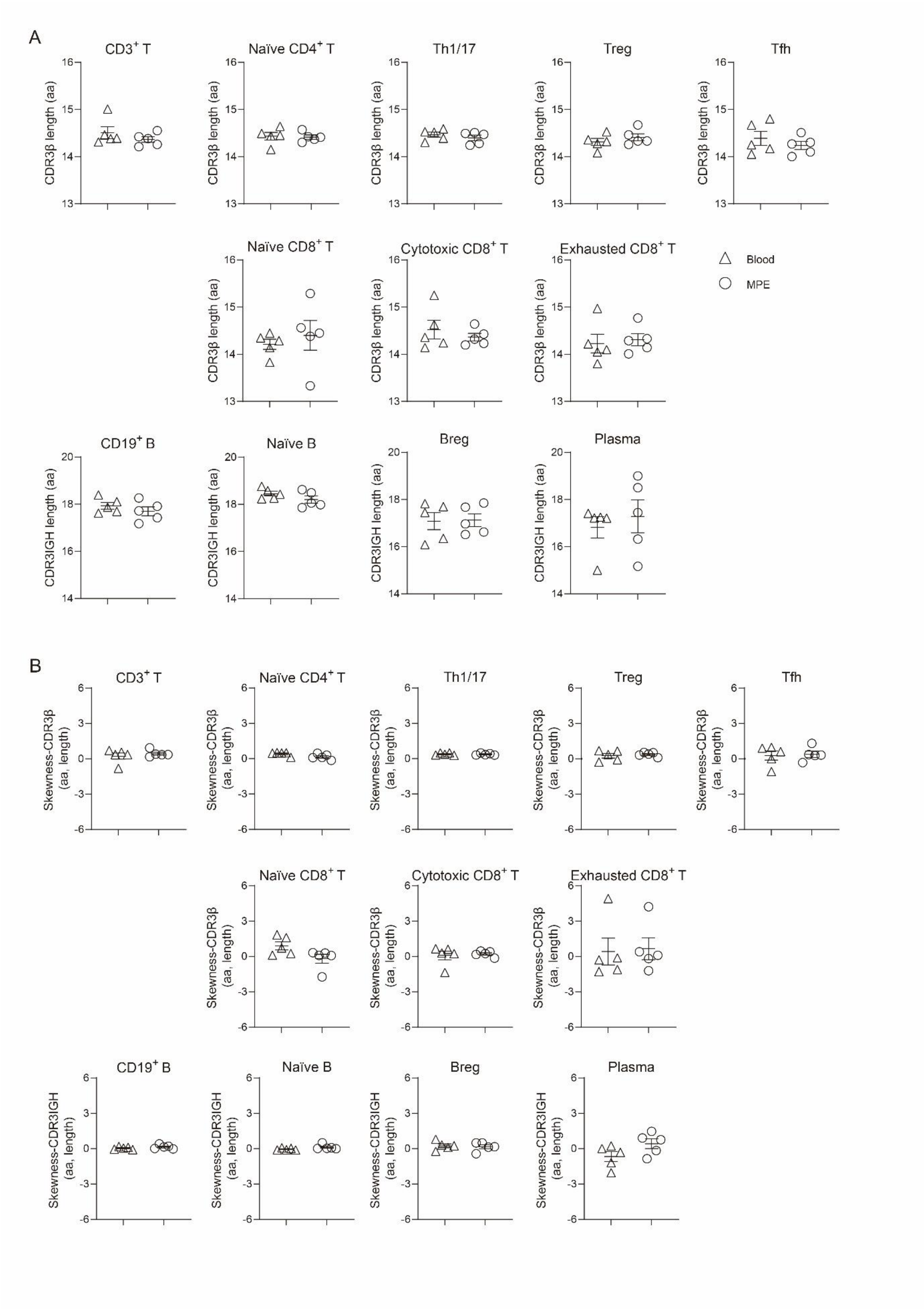

A
CD3+ T
Naïve CD4+ T
Th1/17
Treg
Tfh
CDR3β length (aa)
Naïve CD8+ T
Cytotoxic CD8+ T
Exhausted CD8+ T
Blood
MPE
CD19+ B
Naïve B
Breg
Plasma
CDR3IGH length (aa)
B
Skewness-CDR3β (aa, length)
Skewness-CDR3IGH (aa, length)

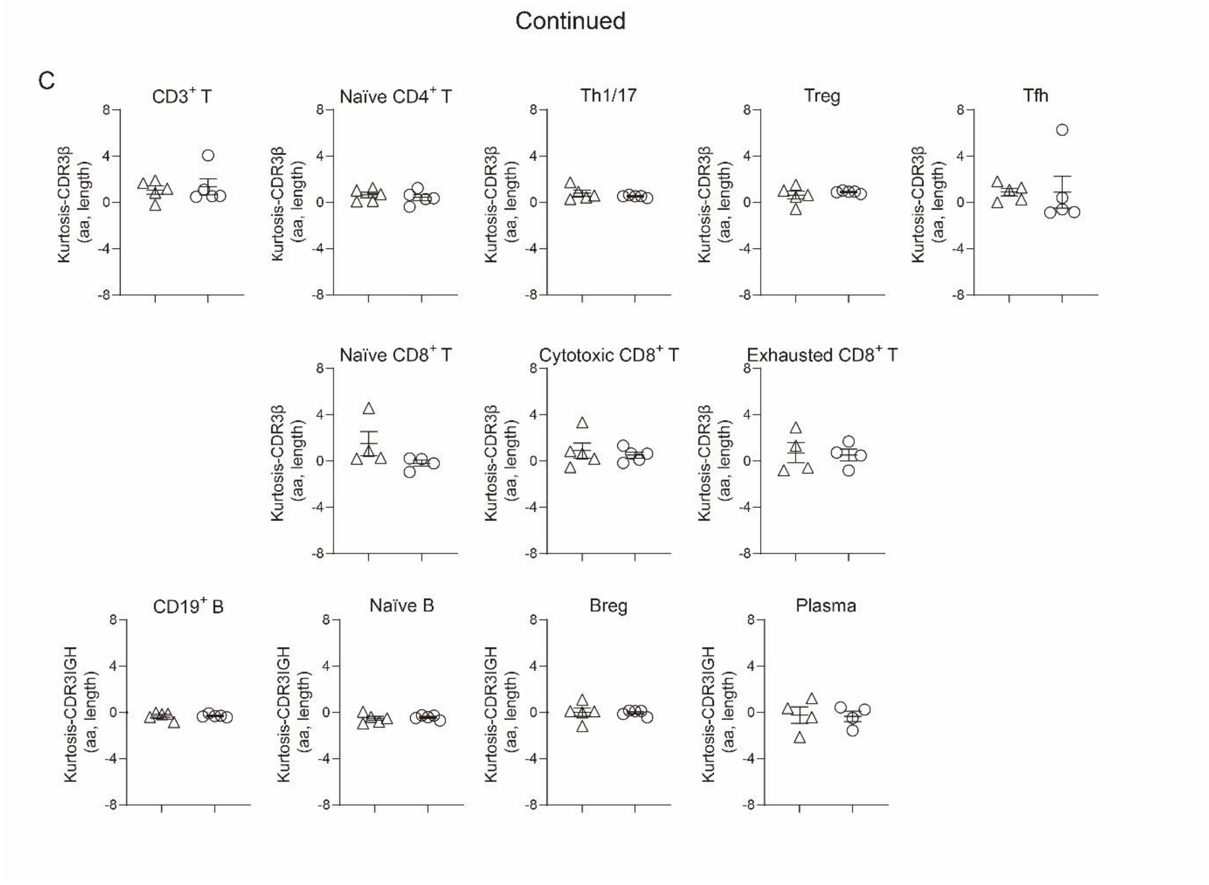

Supplementary Figure 1. Comparisons of the CDR3 sequences in T and B cells between MPE and blood. Average length (A), Kurtosis (B) and Skewness index (C) of length distribution of CDR3 aa sequences. Subset containing three or fewer cells was excluded from the Kurtosis index analysis. Data are presented as means ± SEM. All $P > 0.05$, determined by paired t test.

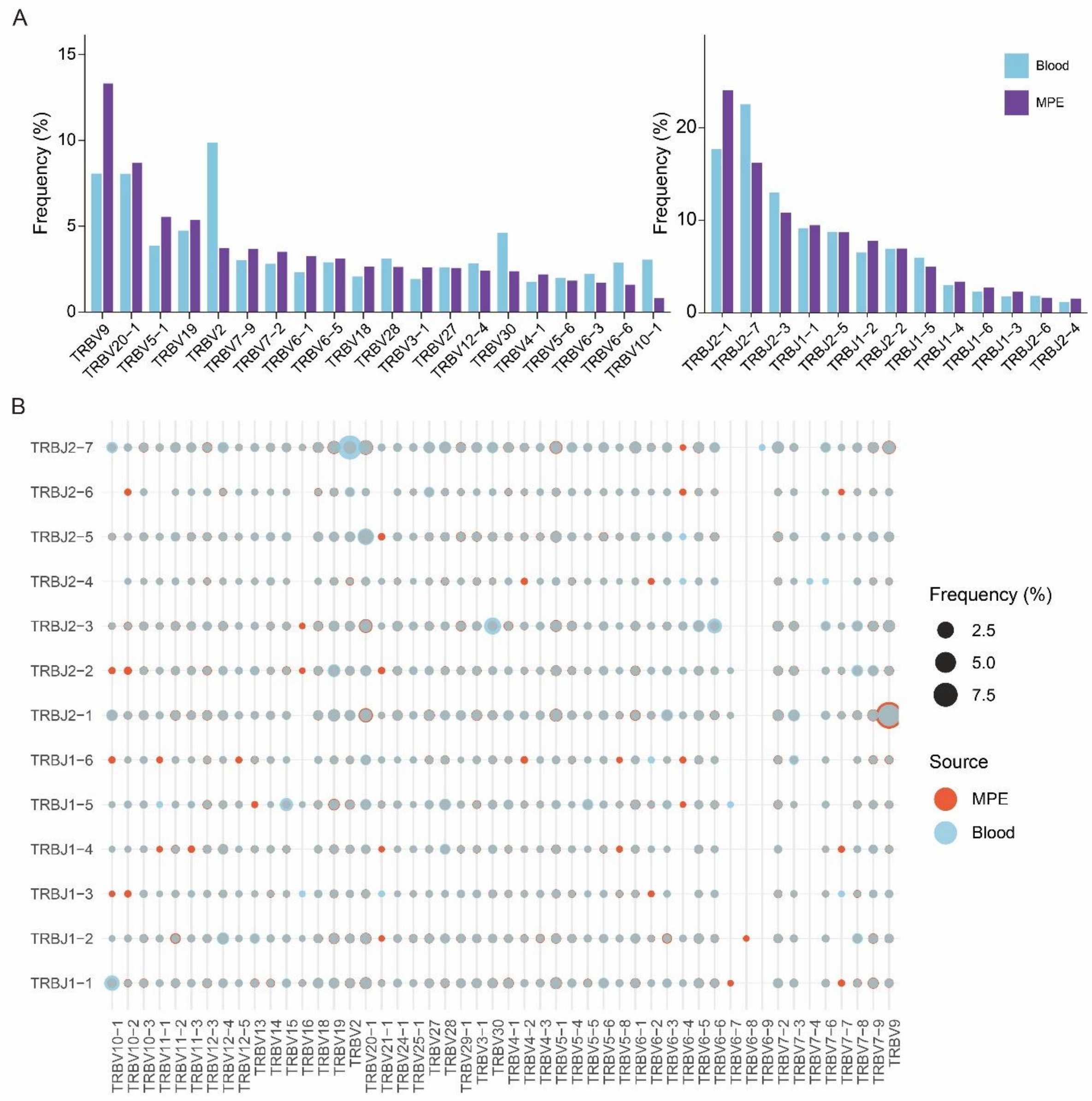
A
Frequency (%)
0
5
10
15
TRBV9
TRBV20−1
TRBV5−1
TRBV19
TRBV2
TRBV7−9
TRBV7−2
TRBV6−1
TRBV6−5
TRBV18
TRBV28
TRBV3−1
TRBV27
TRBV12−4
TRBV30
TRBV4−1
TRBV5−6
TRBV6−3
TRBV6−6
TRBV10−1
Frequency (%)
0
10
20
TRBJ2−1
TRBJ2−7
TRBJ2−3
TRBJ1−1
TRBJ2−5
TRBJ1−2
TRBJ2−2
TRBJ1−5
TRBJ1−4
TRBJ1−6
TRBJ1−3
TRBJ2−6
TRBJ2−4
Blood
MPE
B
TRBJ2−7
TRBJ2−6
TRBJ2−5
TRBJ2−4
TRBJ2−3
TRBJ2−2
TRBJ2−1
TRBJ1−6
TRBJ1−5
TRBJ1−4
TRBJ1−3
TRBJ1−2
TRBJ1−1
TRBV10−1
TRBV10−2
TRBV10−3
TRBV11−1
TRBV11−2
TRBV11−3
TRBV12−3
TRBV12−4
TRBV12−5
TRBV13
TRBV14
TRBV15
TRBV16
TRBV18
TRBV19
TRBV2
TRBV20−1
TRBV21−1
TRBV24−1
TRBV25−1
TRBV27
TRBV28
TRBV29−1
TRBV3−1
TRBV30
TRBV4−1
TRBV4−2
TRBV4−3
TRBV5−1
TRBV5−4
TRBV5−5
TRBV5−6
TRBV5−8
TRBV6−1
TRBV6−2
TRBV6−3
TRBV6−4
TRBV6−5
TRBV6−6
TRBV6−7
TRBV6−8
TRBV6−9
TRBV7−2
TRBV7−3
TRBV7−4
TRBV7−6
TRBV7−7
TRBV7−8
TRBV7−9
TRBV9
Frequency (%)
2.5
5.0
7.5
Source
MPE
Blood

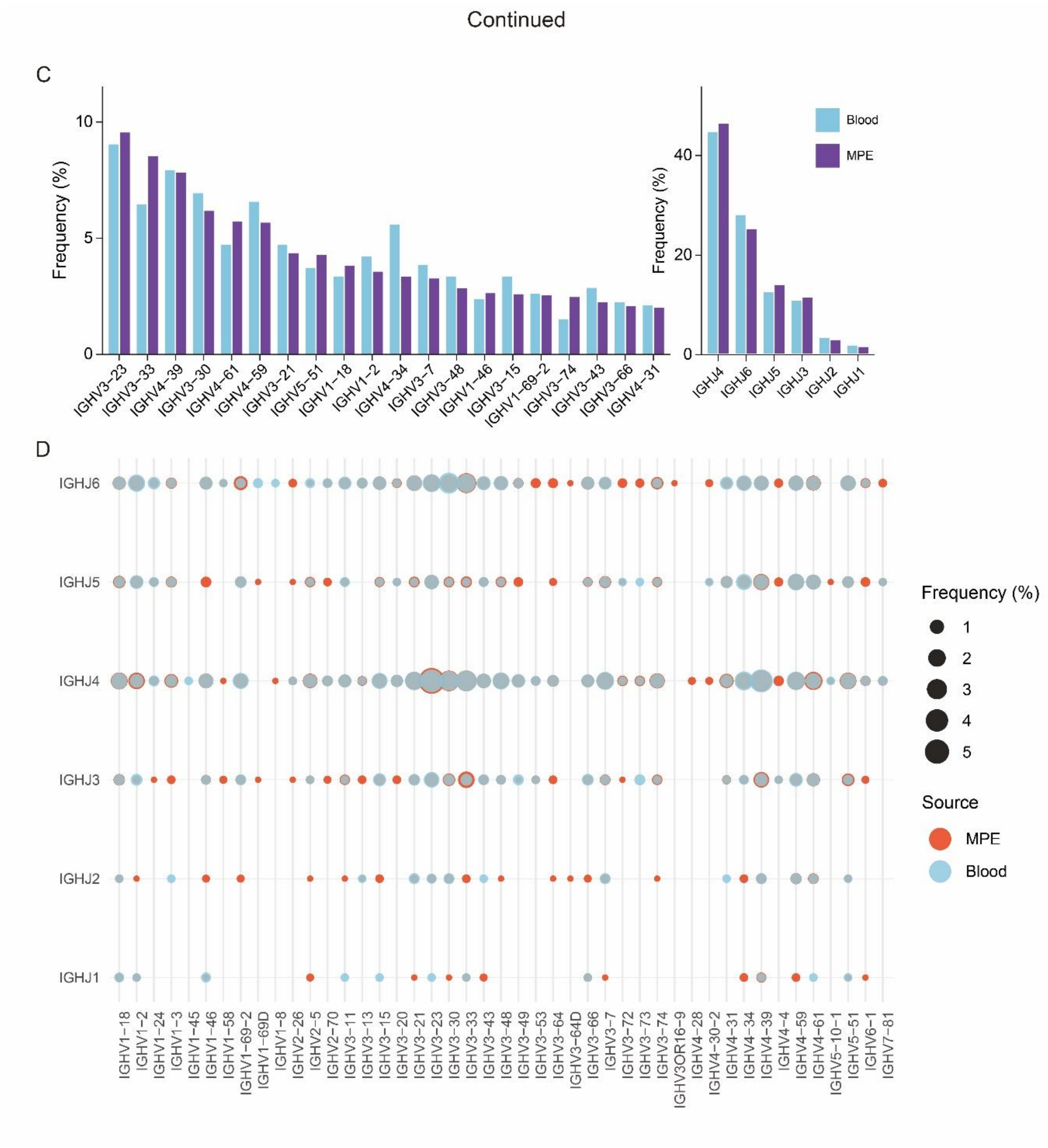

Supplementary Figure 2. TRB and IGH V and J gene segment usage in MPE and paired blood samples, ordered by frequency in MPE. (A) Frequencies of the top 20 TRBV and TRBJ gene segments in T cells. (B) Frequencies of TRBV–TRBJ gene combinations in T cells. (C) Frequencies of the top 20 IGHV and IGHJ gene segments in B cells. (D) Frequencies of IGHV–IGHJ gene combinations in B cells.

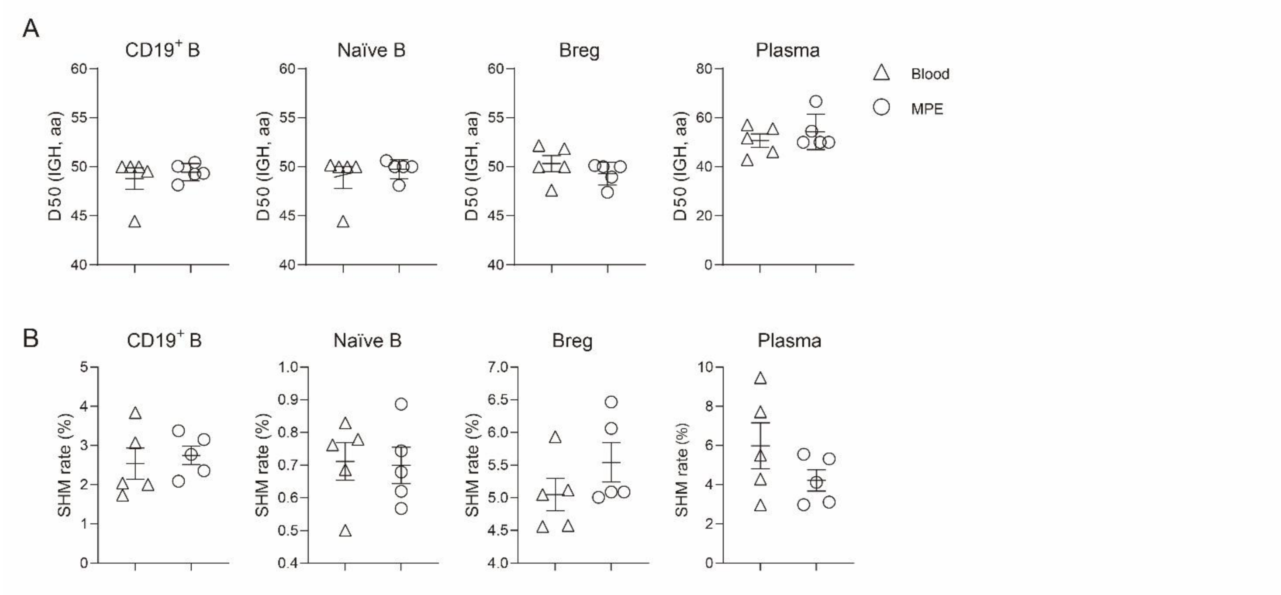

Supplementary Figure 3. Analysis of BCR repertoire diversity and somatic hypermutation across B cell subsets from MPE and Blood. (A) The D50 index (IGH, aa) of BCR clonal diversity across B- cell subsets. (B) The somatic hypermutation rate of BCR sequences across B- cell subsets. No statistically significant differences were observed between the two groups for all subsets (all $P > 0.05$, Mann-Whitney U test).

Supplementary Table 1. Metric information for single-cell BCR sequencing in five human MPE samples*

Patient	P31		P52		P103
Sample type	MPE	Blood	MPE	Blood	MPE
Reads pairs	42,380,989	46,761,066	38,833,123	44,451,084	38,101,140
Estimated Number of Cells	409	198	151	174	1,540
Mean Read Pairs per Cell	103,621	236,167	257,173	255,466	24,741
Number of Cells With Productive V-J Spanning Pair	358	165	136	159	1,409
Reads Mapped to Any V(D)J Gene, %	90.7	89.0	83.7	82.6	80.2
Reads Mapped to IGH, %	25.6	25.3	24.9	26.2	29.0
Reads Mapped to IGK, %	28.0	51.0	39.9	19.1	28.4
Reads Mapped to IGL, %	32.7	7.8	12.4	30.7	14.0
Median IGH UMIs per Cell	15.0	17.0	18.0	17.0	16.0
Median IGK UMIs per Cell	26.0	50.0	29.0	40.0	25.0
Median IGL UMIs per Cell	24.0	38.0	30.0	31.0	24.0
Cells With Productive V-J Spanning Pair, %	87.5	87.3	90.1	91.4	91.5
Cells With Productive V-J Spanning (IGK, IGH) Pair, %	51.1	54.0	53.6	56.3	60.0
Cells With Productive V-J Spanning (IGL, IGH) Pair, %	38.4	33.9	37.7	36.8	33.9
Paired Clonotype Diversity	315.7	111.1	121.3	105.8	1,366.0
Cells With IGH Contig, %	96.3	93.1	98.7	98.3	96.4
Cells With IGK Contig, %	60.9	65.6	62.9	63.8	67.0
Cells With IGL Contig, %	44.0	39.2	45.7	42.5	39.5
Cells With CDR3-annotated IGH Contig, %	91.0	89.4	93.4	94.3	93.4
Cells With CDR3-annotated IGK Contig, %	57.5	60.3	57.6	59.2	63.4
Cells With CDR3-annotated IGL Contig, %	41.3	34.4	40.4	37.9	36.0
Cells With V-J Spanning IGH Contig, %	92.7	88.4	96.0	94.3	94.2
Cells With V-J Spanning IGK Contig, %	58.9	60.8	60.9	60.3	64.6
Cells With V-J Spanning IGL Contig, %	42.8	36.5	43.0	39.7	38.1
Cells With Productive IGH Contig,%	89.2	87.8	92.1	93.1	92.7
Cells With Productive IGK Contig,%	57.0	58.7	57.6	58.6	63.1
Cells With Productive IGL Contig,%	40.6	34.4	39.7	37.9	35.6

Continued

Patient	P103	P106		P108	
Sample type	Blood	MPE	Blood	MPE	Blood
Reads pairs	35,819,620	34,126,784	35,302,183	33,080,888	44,223,597
Estimated Number of Cells	479	824	119	284	183
Mean Read Pairs per Cell	74,780	41,416	296,657	116,482	241,659
Number of Cells With Productive V-J Spanning Pair	445	711	79	246	161
Reads Mapped to Any V(D)J Gene, %	82.2	66.1	61.3	16.0	28.6
Reads Mapped to IGH, %	28.1	30.3	23.1	5.3	8.0
Reads Mapped to IGK, %	35.2	14.0	19.6	7.9	13.8
Reads Mapped to IGL, %	11.3	10.4	6.0	2.6	6.6
Median IGH UMIs per Cell	22.0	15.0	13.0	12.0	19.0
Median IGK UMIs per Cell	52.0	26.0	36.0	24.0	52.0
Median IGL UMIs per Cell	38.5	18.0	28.0	22.0	36.5
Cells With Productive V-J Spanning Pair, %	92.9	86.3	66.4	86.6	88.0
Cells With Productive V-J Spanning (IGK, IGH) Pair, %	57.8	55.5	41.2	52.5	49.7
Cells With Productive V-J Spanning (IGL, IGH) Pair, %	35.5	31.7	26.1	34.5	39.3
Paired Clonotype Diversity	435.1	518.4	72.4	230.0	128.3
Cells With IGH Contig, %	98.1	97.7	85.7	90.1	89.1
Cells With IGK Contig, %	62.4	64.1	63.0	66.9	59.6
Cells With IGL Contig, %	41.3	38.6	29.4	45.8	53.0
Cells With CDR3-annotated IGH Contig, %	94.8	91.3	68.1	88.7	89.1
Cells With CDR3-annotated IGK Contig, %	60.8	60.9	44.5	61.3	56.3
Cells With CDR3-annotated IGL Contig, %	37.4	35.6	28.6	40.8	49.2
Cells With V-J Spanning IGH Contig, %	96.7	93.4	68.9	88.7	88.5
Cells With V-J Spanning IGK Contig, %	61.8	61.5	44.5	62.7	58.5
Cells With V-J Spanning IGL Contig, %	39.2	37.3	28.6	42.6	49.2
Cells With Productive IGH Contig,%	94.4	89.7	68.1	87.7	88.5
Cells With Productive IGK Contig,%	60.5	60.2	43.7	60.6	54.1
Cells With Productive IGL Contig,%	37.0	34.7	28.6	38.7	46.4

*MPE, malignant pleural effusion.

Supplementary Table 2. Analyzed cell numbers with in each subset from five MPE patients*

Patient ID	P31		P52		P103		P106		P108		Total	
Sample type	MPE	Blood	MPE	Blood	MPE	Blood	MPE	Blood	MPE	Blood	MPE	Blood
T cell	2,696	1,410	4,229	2,260	4,958	2,761	6,329	4,681	5,320	2,365	23,532	13,477
CD4^{+} T	2,233	631	1,291	1,008	4,271	1,957	4,253	1,447	4,179	1,447	16,227	6,490
Naïve CD4	256	124	67	210	3,702	1,092	628	813	953	745	5,606	2,984
Th1/17	1,634	390	1,052	704	337	741	2,448	518	2,494	545	7,965	2,898
Treg	320	70	139	72	223	92	467	40	674	111	1,823	385
Tfh	23	47	33	22	9	32	710	76	58	46	833	223
CD8^{+} T	463	776	2,937	1,252	686	804	2,074	3,234	1,141	917	7,301	6,983
Naïve CD8	17	11	3	6	244	186	62	65	221	214	547	482
Cytotoxic CD8	381	742	713	542	433	613	1,850	3,080	896	673	4,273	5,650
Exhausted CD8	65	23	2,221	704	9	5	162	89	24	30	2,481	851
Proliferated T	0	3	1	0	1	0	2	0	0	1	4	4
B cell	300	128	113	110	1,171	390	586	68	201	113	2,371	809
Naïve B	201	96	81	80	730	281	171	34	83	59	1,266	550
Breg	96	23	26	22	413	80	404	27	112	40	1,051	192
plasma	3	9	6	8	28	29	11	7	6	14	54	67

*MPE, malignant pleural effusion.

Supplementary Table 3. S%TGGNTE, SLG%E, SLTGG%TE, and S%GET pattern clusters of similar CDR3 by Grouping of Lymphocyte Interactions by Paratope Hotspots version 2*

Pattern	Fisher _score	Number _subject	Number _unique_CDR3	Type	TRB	V	J
S%TGGNTE	0.0048	5	4	global-S%TGGNTE	CASSLTGGNTEAFF	TRBV7-9	TRBJ1-1
S%TGGNTE	0.0048	5	4	global-S%TGGNTE	CASSLTGGNTEAFF	TRBV7-9	TRBJ1-1
S%TGGNTE	0.0048	5	4	global-S%TGGNTE	CASSLTGGNTEAFF	TRBV14	TRBJ1-1
SLG%E	0.0036	5	11	global-SLG%E	CASSLGSEAFF	TRBV12-3	TRBJ1-1
SLG%E	0.0036	5	11	global-SLG%E	CASSLGSEAFF	TRBV12-3	TRBJ1-1
SLTGG%TE	0.0021	5	4	global-SLTGG%TE	CASSLTGGNTEAFF	TRBV7-9	TRBJ1-1
SLTGG%TE	0.0021	5	4	global-SLTGG%TE	CASSLTGGNTEAFF	TRBV7-9	TRBJ1-1
SLTGG%TE	0.0021	5	4	global-SLTGG%TE	CASSLTGGNTEAFF	TRBV14	TRBJ1-1
S%GET	0.0046	5	8	global-S%GET	CASSLGETQYF	TRBV5-6	TRBJ2-5
S%GET	0.0046	5	8	global-S%GET	CASSLGETQYF	TRBV5-1	TRBJ2-5
S%GET	0.0046	5	8	global-S%GET	CASSLGETQYF	TRBV13	TRBJ2-5
S%GET	0.0046	5	8	global-S%GET	CASSLGETQYF	TRBV7-9	TRBJ2-5
S%GET	0.0046	5	8	global-S%GET	CASSLGETQYF	TRBV7-2	TRBJ2-5
S%GET	0.0046	5	8	global-S%GET	CASSLGETQYF	TRBV7-2	TRBJ2-5
S%GET	0.0046	5	8	global-S%GET	CASSLGETQYF	TRBV7-6	TRBJ2-5

*%, amino acid positions in the TCRs within the clusters that are available for substitution; global, global similarity.
